# Simultaneous quantitation of 17 endogenous adrenal corticosteroid hormones in human plasma by UHPLC-MS/MS and their application in congenital adrenal hyperplasia screening

**DOI:** 10.3389/fchem.2022.961660

**Published:** 2022-08-11

**Authors:** Qiaoxuan Zhang, Min Zhan, Huihui Wu, Pinning Feng, Xing Jin, Zemin Wan, Jun Yan, Pengwei Zhang, Peifeng Ke, Junhua Zhuang, Jiuyao Zhou, Liqiao Han, Xianzhang Huang

**Affiliations:** ^1^ Department of Laboratory Medicine, The Second Affiliated Hospital of Guangzhou University of Chinese Medicine (Guangdong Provincial Hospital of Chinese Medicine), Guangzhou, China; ^2^ Anhui Prevention and Treatment Center for Occupational Disease, Anhui No. 2 Provincial People’s Hospital, Hefei, China; ^3^ Department of Laboratory Medicine, Sun Yat-Sen University First Affiliated Hospital, Guangzhou, China; ^4^ The Affiliated Hospital of Yangzhou University, Yangzhou University, Yangzhou, China; ^5^ Department of Pharmacology, School of Pharmaceutical Sciences, Guangzhou University of Chinese Medicine, Guangzhou, China

**Keywords:** UHPLC-MS/MS, adrenal hormones, simultaneous quantitation, congenital adrenal hyperplasia, diagnostic efficiency

## Abstract

Accurate investigation of adrenal hormone levels plays a vital role in pediatric endocrinology for the detection of steroid-related disorders. This study aims to develop a straightforward, sensitive UHPLC-MS/MS method to quantify 17 endogenous adrenal corticosteroid hormones in human plasma. These hormones are the main ingredients in the synthetic and metabolic pathways of adrenal corticosteroid hormones. Chromatographic separation was achieved on a C18 column before electrospray ionization triple-quadrupole mass spectrometry in multiple reaction monitoring mode with a run time of 7 min. The samples were extracted by liquid-liquid extraction and required no derivatization. Analytical performance was evaluated, including linearity, analytical sensitivity, accuracy, precision, and specificity. Plasma specimens from 32 congenital adrenal hyperplasia (CAH) patients and 30 healthy volunteers were analyzed to further reveal the diagnostic value of multiple steroid hormones in the synthetic and metabolic pathways of adrenal corticosteroid in CAH diagnosis. All hormones were effectively extracted and separated using our method. The method was essentially free from potential interference of isomers or structural analogues. The imprecisions were <10%. The lower limits of quantification varied from 0.05 to 15.0 ng/ml. Good linearity coefficients (*r* ^*2*^ > 0.998) were also obtained for most hormones in the required concentration range, except for 21-deoxycortisol (*r* ^*2*^ = 0.9967) and androstenediol (*r* ^*2*^ = 0.9952). The recoveries for the steroid hormones ranged from 91.7 to 109.8%. We developed the UHPLC-MS/MS method for the simultaneous measurement of steroid hormones. The results showed that measurement of steroid hormones simultaneously could improve the diagnostic efficiency of CAH.

## 1 Introduction

Congenital adrenal hyperplasia (CAH) comprises a group of autosomal recessive disorders caused by deficient adrenal corticosteroid biosynthesis (White and Speiser, 2000; Merke and Bornstein, 2005). It is caused by defects in one of the steroidogenic enzymes involved in cortisol biosynthesis or in the electron providing factor, P450 oxidoreductase (POR). 21-hydroxylase and 11β-hydroxylase deficiency are the most frequent forms of CAH (White and Speiser, 2000). Congenital lipoid adrenal hyperplasia, resulting from steroidogenic acute regulatory protein (StAR) deficiency affecting mitochondrial cholesterol uptake, is a subtype of the disease complex with the unique characteristics of lipid accumulation leading to cell destruction (Ishii et al., 2016). In each case, negative feedback inhibition of cortisol is reduced and adrenal hormone and androgen secretion are altered depending on the steroid-producing pathway involved. With the different disease types, the changes in hormone levels were significantly different.

**FIGURE 1:**
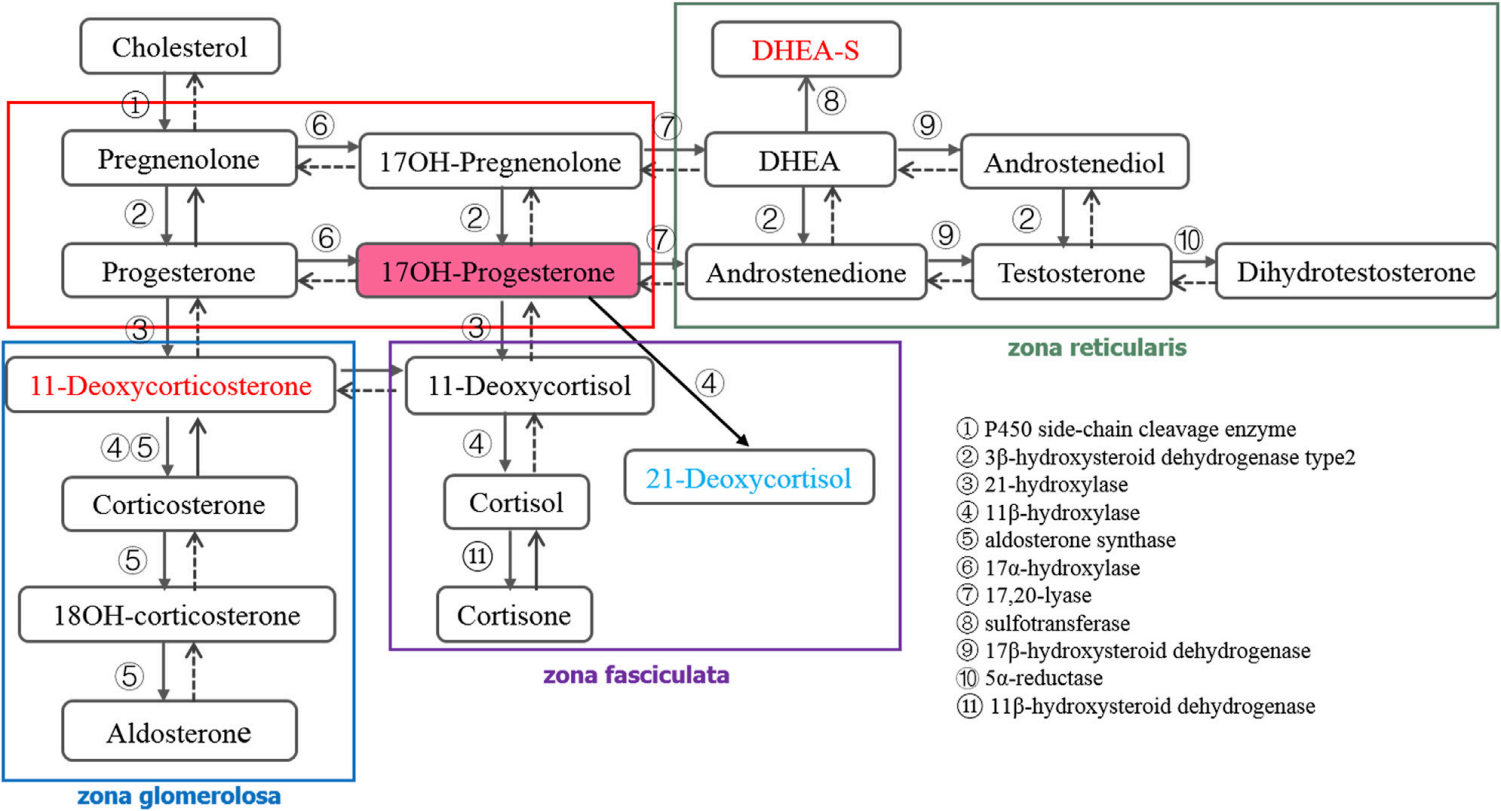
Synthetic and metabolic pathways of adrenal corticosteroid hormones.

Steroid hormones are highly potent substances catalyzed by a series of enzymes from cholesterol, mainly divided into adrenal corticosteroid hormones (Figure 1), sex hormones, and vitamin D. The level of various steroid hormones is extremely low *in vivo*, and the concentration level is in the order of nmol/L or pmol/L (Rauh, 2010). Measurement of steroid hormones in the clinical setting poses a number of difficulties for analytical methods. The assays need to be sensitive, specific, accurate, and precise over a wide concentration range. Despite the widespread importance of steroid hormones in human biology, our ability to measure them properly has not kept pace with their increasing importance in clinical medicine and research (Rosner and Vesper, 2008). Therefore, developing a reliable and specific analysis method has become the primary task of clinical disease diagnosis.

At present, the quantitation of steroid hormones mainly includes immunoassay and mass spectrometry (MS) methods (Taieb et al., 2002; Speiser, 2004; Olisova et al., 2019). As an approach, with simple and rapid operation, immunoassay-based methods have been routinely used for the measurement of plasma biomolecules in clinical settings, but nonspecific interaction remains a challenge for these methods. For instance, 17α-hydroxyprogesterone (17OHP), 11-deoxycorticosterone (DOC), and steroids were found to interfere with the measurement of progesterone in the immunoassay (Wilson et al., 1998). Meanwhile, immunoassays cannot simultaneously measure multiple hormones in the same specimen. Therefore, efficient, in-depth, and accurate study of steroid metabolism profile cannot be achieved (Taieb et al., 2002).

The purpose of a newborn screening program is to diagnose congenital diseases accurately, such as CAH, as early as possible before symptoms appear (Witchel, 2019). However, the immunoassay commonly used often produces false positive results, which increases the psychological burden and economic pressure on patients. LC-MS/MS has shown great potential in the clinical diagnosis of CAH because of its high sensitivity, high selectivity, and wide dynamic range (Zhang et al., 2017; Jeong et al., 2021). In order to improve the positive predictive value of CAH screening, the endocrine society expert group released the 21-hydroxylase deficiency clinical practice guideline in 2018 (second edition) (Speiser et al., 2018). Compared with the first edition published in 2010 (Speiser et al., 2010) recommended that measurement of 17OHP concentration by routine method in screening newborn, and also recommended that the preferred secondary screening be performed by LC-MS/MS. Despite the elevation of 17OHP level being mostly considered as the primary indicator for determining 21-hydroxylase deficiency, other forms of CAH may also lead to the change of 17OHP level (Schwarz et al., 2009). Additionally, it can also result in the change of other steroid hormones in the synthetic and metabolic pathways (Figure 1). Janzen et al. (2007) established that LC-MS/MS not only avoided the problem of high false positives in the diagnosis of CAH by immunoassay, but also found that the sensitivity of LC-MS/MS can be further improved by calculating the value of (17OHP+21-deoxycortisol)/cortisol. Therefore, more attention should be paid to the changes of other hormone levels during secondary screening, and appropriate calculation formulas should be selected to improve the sensitivity of detection methods, which also provides a new idea for the diagnosis of CAH (Ambroziak et al., 2016). LC-MS/MS was used to measure the adrenal corticosteroid hormones in the synthetic and metabolic pathways simultaneously, which has great significance for the diagnosis of CAH or other diseases related to hormone metabolism.

To better exploit the importance of steroid hormones for CAH, a simple, accurate, sensitive, and specific isotope-dilution UHPLC-MS/MS method was developed for the simultaneous quantitation of 17 endogenous steroid hormones in human plasma in this study. The method has been optimized and the performance has been systematically evaluated. The optimized UHPLC-MS/MS method was applied to measure 17 endogenous adrenal corticosteroid hormones in the plasma of CAH patients and healthy volunteers.

## 2 Experimental

### 2.1 Materials and instruments

Aldosterone, 18-hydroxycorticosterone (18OH-corticosterone), cortisol, 11-deoxycorticosterone (DOC), testosterone, progesterone, pregnenolone, 17α-hydroxyprogesterone (17OHP), [9,11,12,12-D4]18OH-corticosterone, [9,11,12,12-D4]corticosterone, [2,2,3,4,4-D5]dehydroepiandrosterone (DHEA), [20,21-13C2-16,16-D2]pregnenolone, and ammonium fluoride were obtained from Sigma-Aldrich (Shanghai, China). Corticosterone, androstenedione, and dihydrotestosterone (DHT) were purchased from Dr. Ehrensorfer (Germany). 11-deoxycortisol, 17OH-pregnenolone, 16OHP, [2,2,4,6,6,21,21-D7]aldosterone, [2,2,4,6,6,9,12,12-D8]cortisone, [2,2,4,6,6,21,21-D7]11-deoxycorticosterone, and [21,21,21-D3]17OH-pregnenolone were obtained from TRC (Toronto, Canada). [9,11,12,12-D4]cortisol, [2,2,4,6,6-D5]11-deoxycortisol, [2,3,4-^13^C_3_]androstenedione, [16,16,17-D3]testosterone, [16,16,17-D3]DHT, and [2,2,4,6,6,17,21,21,21-D9]progesterone were obtained from Cerilliant (United States). 11OHP was obtained from Aladdin (Shanghai, China). [2,2,4,6,6,21,21,21-D_8_]17OHP was purchased from C/D/N IsotopesInc (Quebec, Canada). DHEA was obtained from Meilunbio (Dalian, China) and cortisone was from Bepure (Beijing, China). 21-deoxycortisol was obtained from Supelco (Shanghai, China). Androstenediol was obtained from Merck (Shanghai, China). LC-MS grade ethyl acetate ethanol, *n*-hexane, methanol, and ethanol were obtained from Merck (Darmstadt, Germany). Water used in the experiments was produced by Simplicity^®^ water purification system from Millipore (MA, United States). Charcoal stripped plasma is a low level steroid plasma. Through carbon adsorption it can reduce the concentration of many hormones and growth factors in plasma, which is obtained by using activated carbon adsorption treatment on the remaining specimens of clinical tests.

ACQUITY UPLC^®^ BEH, C18, 1.7 μm, 50 mm × 2.1 mm column was obtained from Waters (Milford, United States). Polypropylene tubes were purchased from Kirgen Bioscience (Shanghai, China). The following equipment was calibrated by the South China National Center of Metrology (Guangzhou, China): pipettes from Eppendorf Research (Hamburg, Germany); volumetric flasks of BRAND (Germany); and analytical balance (resolution 0.01 mg) from Sartorius Competence (Gottingen, Germany).

### 2.2 Clinical samples

The remaining specimens of clinical tests were collected in this study. All the procedures performed in studies involving human participants were in accordance with the ethical standards of the Ethics Committee of Guangdong Provincial Hospital of Chinese Medicine (ZE2022-064-01). A total of 62 samples (32 CAH patients and 30 healthy volunteers) were assessed in this study. The 32 CAH patients’ samples were from the First Affiliated Hospital of Sun Yat-Sen University. 30 healthy volunteers’ samples were collected from the Guangdong Provincial Hospital of Chinese Medicine. CAH patients were included according to the guidelines for congenital adrenal hyperplasia due to steroid 21-hydroxylase deficiency: an endocrine society clinical practice guideline (Speiser et al., 2018). The basic information on these subjects is summarized in Supplementary Table S1. The collected remaining blood was used for clinical testing in the hospital clinical laboratory. The blood was centrifuged at 3,000 g for 15 min at 4°C with LEGEND MICRO 21R hypothermic centrifugal machine (Thermo Scientific, United States). The supernatant was filled into the numbered 1.5 ml Eppendorf tube in 0.5 ml portions. All the samples were stored immediately in −80°C refrigerator until analysis.

### 2.3 Calibrator, internal standard, and quality control materials preparation

Stock solutions A (Stock A) of the 17 steroids were prepared at 1.0 mg/ml by weighing each standard (accuracy: ±0.1 mg) and dissolving them in methanol separately. Stock solutions B (Stock B) for aldosterone, corticosterone, 11-deoxycortisol, androstenedione, testosterone, 17α-OHP, and DHT were prepared at 20 μg/ml by diluting the corresponding stock A with 50% methanol, as well as 18OH-corticosterone, cortisone, cortisol, 21-deoxycortisol, DOC, androstenediol, 17OH-pregnenolone, progesterone, and pregnenolone prepared at 100 μg/ml. Stock solutions C (Stock C) for 18OH-corticosterone, 21-deoxycortisol, and DOC were prepared at 20 μg/ml by diluting its Stock B with 50% methanol. Working solution 1 (WS1) containing the 17 steroids were prepared at 0.4 μg/ml for aldosterone, 18OH-corticosterone, 21-deoxycortisol, corticosterone, DOC, 11-deoxycortisol, androstenedione, testosterone, 17OHP, and DHT, 1 μg/ml for progesterone and pregnenolone, 6 μg/ml for cortisone, cortisol, androstenediol, and 17OH-pregnenolone, and 60 μg/ml for DHEA by diluting the corresponding stock solutions with 50% methanol. Working solution 2 (WS2) for the 17 steroids was prepared by diluting the WS1 20 times with 50% methanol. The 7-point standard calibration solutions (from ST1 to ST7) were prepared by diluting the WS2 to the final concentrations (ng/ml) of 0.1, 0.5, 1.0, 2.0, 5.0, 10.0, and 20.0 for aldosterone, 18OH-corticosterone, 21-deoxycortisol, corticosterone, DOC, 11-deoxycortisol, androstenedione, testosterone, 17OHP, and DHT; 0.25, 1.25, 2.5, 5.0, 12.5, 25.0, and 50.0 for progesterone and pregnenolone; 1.5, 7.5, 15, 30, 75, 150, and 300 for cortisone, cortisol, androstenediol and 17OH-pregnenolone; and 15, 75, 150, 300, 750, 1,500, and 3,000 for DHEA with 50% methanol.

The same procedure was used to prepare stock and working solutions for the 15 internal standards (ISs) to the final concentrations of 20 ng/ml for 18OH-corticosterone-D_4_, DOC-D_7_, adione-^13^C_3_, 11-deoxycortisol-D_5_, testosterone-D_3_, 17OHP-D_8_, 100 ng/ml for progesterone-D_9_, 200 ng/ml for corticosterone-D_4_ and DHT-D3, 300 ng/ml for cortisone-D_8_ and cortisol-D_4_, 600 ng/ml for aldosterone-D_7_, 1.0 μg/ml for pregnenolone-^13^C_2_-D_2_, 2.0 μg/ml for DHEA-D_5_, and 3.0 μg/ml for 17OH-pregnenolone-D_3_ as IS working solution (ISWS). Stock solutions were stored at −80°C until used, working solutions were stored at 4°C for up to 6 months. All solutions were brought to room temperature for ≥30 min before use to avoid the effects of temperature change on sample volume. Three quality controls (QCs) were prepared by spiking the working standard solution in charcoal-treated human plasma to achieve the final concentrations. During the quantitative process, DHEA-D_5_ was used as IS for androstenediol and corticosterone-D_4_ was used as IS for 21-deoxycortisol. The other hormones were used with corresponding isotope labeled compounds as IS.

### 2.4 Sample preparation

For analysis, blanks, quality controls, and plasma samples (0.2 ml each) were added to 2 ml centrifuge tubes, and mixed vigorously after spiking with 20 μl multiple ISWS to each tube. Then the mixture was equilibrated at room temperature for 30 min followed by liquid-liquid extraction with 1 ml *n-*hexane/ethyl acetate (1:1, v/v). The supernatant was transferred to a new tube after being centrifuged at 15,000 g for 5 min and dried under nitrogen at 45°C, then reconstituted in 200 μl methanol/water (50:50, v/v). 10 μl of each sample was injected for LC-MS/MS analysis.

62 samples from each group were analyzed at random, and QCs were inserted into the analysis sequence to monitor and correct changes in the instrument response. To identify and remove probable characteristic peaks caused by source contaminants, test tube components, or solvent impurities, blank samples were inserted for every ten runs. Analysis was conducted in positive ion mode on plasma samples.

### 2.5 LC-MS/MS conditions

The LC-MS/MS system consisted of a Waters Acquity UPLC™ with a triple quadruple mass detector (Xevo TQ-S), using MassLynx v4.1 software (Waters) for system operation and data acquisition. The desolvation gas was provided by the nitrogen generator (PEAK), while the collision gas was argon. Target steroids were resolved at 45°C on a C18 column (ACQUITY UPLC^®^ BEH, 1.7 μm, 50 mm × 2.1 mm) with 0.2 mM ammonium fluoride (NH_4_F) in water (mobile phase A) and methanol (mobile phase B) at a flow rate of 0.4 ml/min. Separation of the 17 steroids was achieved using a gradient program consisting of an initial condition of 40% mobile phase B for 1 min, then increased from 40 to 55% B over 2.5 min, followed by an increase from 55 to 98% B from 4.5 min to 5.5 min. The column was then re-equilibrated with 40% B from 6.1 min to 7.0 min. The steroid analyses were carried out using electrospray ionization (ESI) in positive ion with multiple reaction monitoring (MRM). The ionization and MS/MS conditions were optimized and summarized as follows: capillary voltage (3.0 kV), source temperature (150°C), desolvation temperature (450°C), cone gas flow (150 L/h), desolvation gas flow (650 L/h), nebulizer (7 Bar), and collision gas flow (0.2 ml/min). Ion transitions and conditions were listed in Table 1. A dwell time of 0.008 s was used for each transition.

**TABLE 1:** Ion transitions and conditions for the 17 steroid hormones and internal standards.

No.	Compound	CAS no.	Molecular weight	Q1 Mass (Da)	Q3 Mass (Da)	Cone (V)	Collision (V)
1-1	Aldosterone	52-39-1	360.44	361.0	343.0	25	15
1-2	Aldosterone-D_7_	1261254-31-2	367.49	368.0	350.0	25	15
2-1	18OH-corticosterone	561-65-9	362.46	363.0	121.1	25	45
2-1	18OH-corticosterone-D_4_	1257742-38-3	366.48	367.0	121.9	25	45
3-1	Cortisone	53-06-5	360.44	361.3	163.1	25	15
3-2	Cortisone-D_8_	1257650-98-8	368.49	369.3	168.2	25	15
4-1	Cortisol	50-23-7	362.46	363.0	121.0	25	45
4-2	Cortisol-D_4_	73565-87-4	366.48	367.0	122.0	25	30
5	21-Deoxycortisol	641-77-0	346.46	347.2	121.1	25	28
6-1	Corticosterone	50-22-6	346.46	347.3	329.0	25	10
6-2	Corticosterone-D_4_	1261254-51-6	350.49	351.3	333.0	25	10
7-1	11-Deoxycortisol	152-58-9	346.46	347.2	97.0	25	20
7-2	11-Deoxycortisol-D_5_	1258063-56-7	351.49	352.2	100.0	25	26
8-1	Androstenedione	63-05-8	286.41	287.2	97.0	25	26
8-2	Androstenedione-^13^C_3_	327048-86-2	289.39	290.2	100.0	25	26
9-1	11-Deoxycorticosterone (DOC)	64-85-7	330.46	331.3	97.0	25	26
9-2	DOC-D_7_	—	337.44	338.4	100.0	25	26
10-1	Testosterone	58-22-0	288.42	289.4	97.0	25	28
10-2	Testosterone-D_3_	77546-39-5	291.44	292.2	97.0	25	28
11	Androstenediol	521-17-5	290.44	255.3	159.3	25	25
12-1	Dehydroepiandrosterone (DHEA)	53-43-0	288.43	289.2	253.0	25	10
12-2	DHEA-D_5_	97453-25-3	293.46	294.3	258.2	25	14
13-1	17α-Hydroxyprogesterone (17OHP)	604-09-1	330.46	331.4	97.0	25	26
13-2	17OHP -D_8_	850023-80-2	338.46	339.4	100.1	25	26
14-1	17OH-pregnenolone	387-79-1	332.48	333.3	297.2	25	10
14-2	17OH-pregnenolone-D_3_	105078-92-0	335.48	336.1	300.1	25	10
15-1	DHT	521-18-6	290.44	291.4	255.0	25	10
15-2	DHT-D_3_	79037-34-6	293.46	294.4	258.2	25	10
16-1	Progesterone	57-83-0	314.46	315.4	97.0	25	45
16-2	Progesterone-D_9_	15775-74-3	323.52	324.4	100.0	25	27
17-1	Pregnenolone	145-13-1	316.48	317.2	281.3	25	13
17-2	Pregnenolone-^13^C_2_-D_2_	—	320.48	321.2	285.3	25	13

### 2.6 Method validation

The performance of LC-MS/MS method for the 17 steroids was validated for imprecision, accuracy, linearity, and sensitivity. The total imprecision was assessed by analyzing three levels of plasma-pool samples in triplicate over 5 days according to CLSI document EP10-A3 (Clinical and Laboratory Standards Institute, 2014). Recovery experiment was used for accuracy assessment using charcoal stripped plasma, which spiked standards at low, medium, and high levels for each steroid. The linearity was evaluated for each steroid by serial dilution of the plasma sample according to CLSI EP6-A (Clinical and Laboratory Standards Institute, 2003). Sensitivity of the method was assessed by the lower limit of detection (LoD) and lower limit of quantitation (LLoQ). The LoD was defined for each steroid at the concentration at which the signal to noise (S/N) ratio was ≥3. LLoQ was defined as the concentration at which the S/N ratio was ≥10 and CV ≤ 20.0% (*n* = 4). Specificity and carry-over have also been evaluated.

### 2.7 Data analysis

SPSS 20.0, MedCalc statistical, Origin85, GraphPad Prism 5, and Microsoft Excel 2010 software programs were used for data analysis. For the peak area of interest and data acquisition, Waters MassLynx version 4.1 SCN876 was used and the accuracy for defining the peak area was verified by a technician. Each calibration curve was built into each measurement series. Linear regression with 1/x weighting was used as a curve fit by combining the data for calibrators which run before and after the samples.

Prior to statistical analysis, the concentrations of 17 hormones in plasma detected by LC-MS/MS were sorted into the same unit. The principal components analysis (PCA) model and partial least squares discriminate analysis (PLS-DA) model were performed by SIMCA 13.0 software (Umetrics, Umeå, Sweden). Response permutation test was performed 200 times to evaluate whether the model had over fitting. Potential biomarkers were extracted from constructed loading plots, followed by analysis with PLS-DA, and the biomarkers were chosen based on the VIP value (variable importance in the projection). Other statistical analyses were performed using SPSS 20.0 (SPSS, Chicago, United States). Significant differences of metabolites were considered when *p* values of *t* test were less than 0.05. And to estimate the diagnostic abilities of biomarkers, systematic cluster analysis, ROC curve, and the binary logistic regression were made by using SPSS 20.0.

## 3 Results and discussion

There are 18 kinds of steroid hormones involved in the synthetic and metabolic pathways of adrenal corticosteroids. The metabolism and transformation of these hormones are regulated by different enzymes and play an important role in indicating the diseased state of the body. Comprehensive and accurate measurement of these hormones will help to diagnose clinical diseases and discover potential pathogenic factors. Therefore, we want to develop an UHPLC-MS/MS method to quantify these 18 hormones simultaneously. Counterproductively, DHEA-S, as a sulfonate of DHEA, differs greatly from other hormones in its physical and chemical properties. As a result, it differs from other hormones in sample pretreatment and ion mode selection. Therefore, in order to improve the overall performance of the method, we abandoned DHEA-S in the method development.

### 3.1 MS condition optimization

**FIGURE 2:**
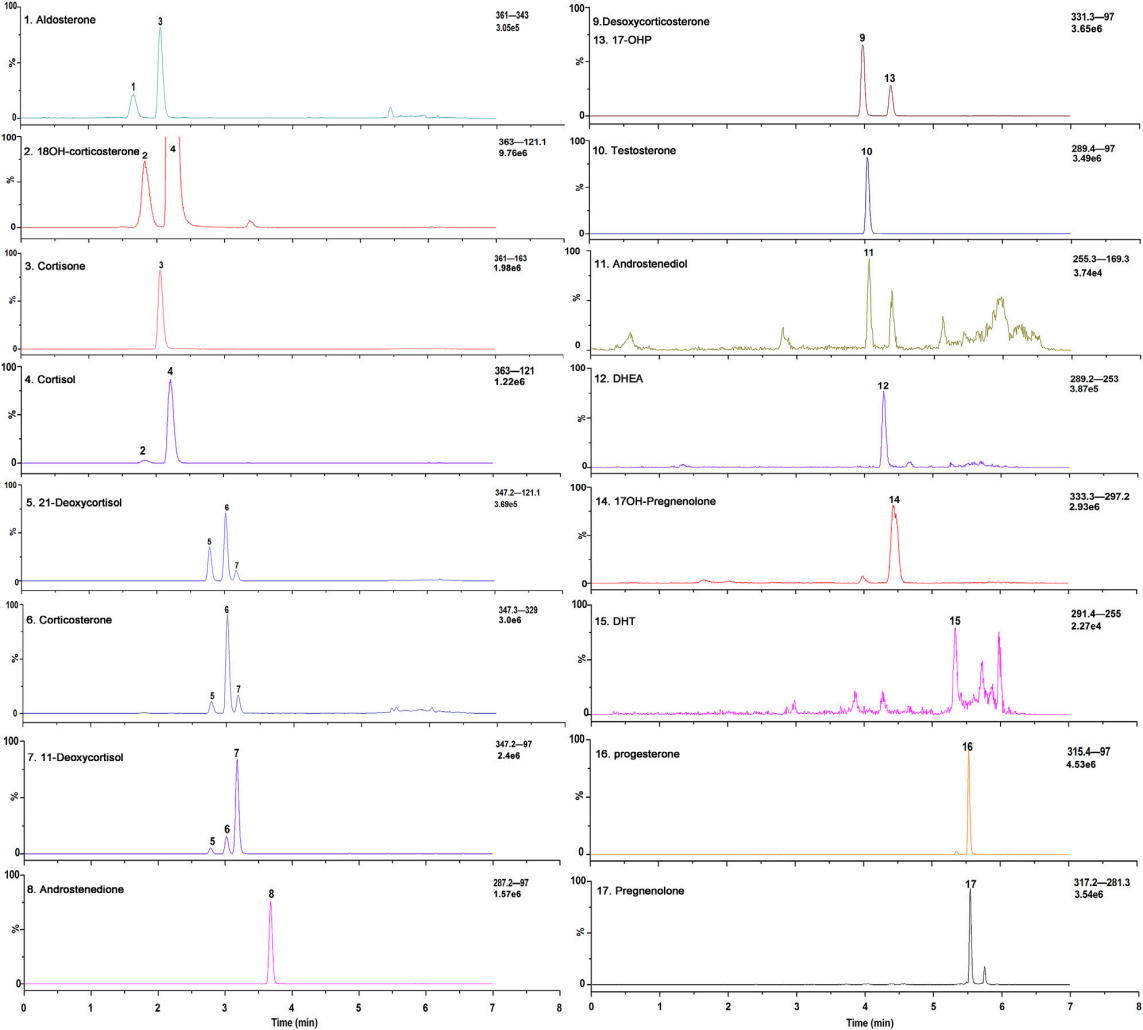
Exemplary selected ion chromatograms of steroid hormones a in a stripped extracted human plasma.

All the parameters were optimized by infusing a 500 ng/ml solution of each steroid hormone and their IS at a flow rate of 7 μl/min. To develop an MRM method, positive ESI mode was used to identify the precursor and product ions for the 17 endogenous steroid hormones (Figure 2). Two product ions were obtained for each steroid hormone: the predominant product ion was used as the quantifier ion and the other ion was used as the qualifier ion. Compound-dependent parameters such as fragment and collision energies were tuned to produce the most intense mass spectrum signals for each analyte, as shown in Table 1.

### 3.2 LC condition optimization

Because of the presence of metabolites or structural analogs that have the same molecular masses as the steroids as well as low concentrations, simultaneous quantitation of steroid hormones in human plasma is a great challenge. In addition to the 17 steroid hormones in this quantitation, a total of 19 steroid hormones, including 11OHP and 16OHP, were tested to determine whether they could interfere with the measurement of each steroid. Among these structural analogs, 11OHP, 16OHP, and DOC have the same molecular mass as 17OHP. The same situation was also observed in 21-deoxycortisol, corticosterone, and 11-deoxycortisol, which had a molecular weight of 346.46. The molecular weight of aldosterone and cortisone was 360.44. The molecular weight of 18OH-corticosterone and cortisol was 362.46. If chromatographic separation from isomers or structural analogues of these species could not be achieved, the MS-based methods would yield biased results. We have optimized several parameters of the chromatographic separation to improve the specificity and sensitivity of the method.

The composition of the mobile phase plays an important role in chromatographic separation. The organic phase (mobile phase B) was optimized by methanol and acetonitrile with or without formic acid. And the results showed that methanol showed better separation ability for the steroid hormones. Additionally, the sensitivity of methanol without formic acid is higher than that with formic acid and use of methanol instead of acetonitrile resulted in superior selectivity with the appropriate gradient elution. Furthermore, the gradient elution was also systematically investigated. After optimization of the flow rate, the initial ratio of the mobile phase, and the slope of the gradient, adequate separation of all the steroid hormones, and their structural analogs was achieved. The final chromatographic separation of all the steroid hormones is shown in Figure 2. The structural analogs with no peaks are undetectable steroid hormones under MS conditions, which indicates that none of the steroids evaluated interfered with the LC-MS/MS method.

The nature and concentration of buffer A were also optimized to enhance the sensitivity of the method. Several ammonium salts, such as ammonium formate, ammonium acetate, and NH_4_F were tested as reported elsewhere (Fiers et al., 2012; Mario et al., 2015; Pesek and Matyska, 2015; Zhang et al., 2021). Use of NH_4_F as a buffer was found to promote the degree of ionization and further enhance the signal strength, which is also consistent with a previous report (Fiers et al., 2012), since addition of some fluoride ions to the mobile phase promoted steroids ionization. While other conditions were kept constant, the concentration of NH_4_F was adjusted to five levels: 0.02, 0.05, 0.1, 0.2, and 0.5 mmol/L. A mixed solution of the steroid hormones (the ST5) was used as the test solution. The results showed that the optimum fluoride concentration was around 0.2 mmol/L, as a trade-off between the range where a low concentration impacts the process yield and a high concentration of fluoride ions starts to hamper analyte ionization because of the ion suppression effect generated by the massive presence of negatively charged fluoride ions (Fiers et al., 2012).

### 3.3 Extraction efficiency

The better detection sensitivity could be achieved by improving the extraction recoveries in certain LC-MS/MS conditions. Our previous research about the reference measurement procedure of steroid hormones has found that the extraction efficiency by using the mixture solution of *n*-hexane/ethyl acetate is higher than previous research’s solid-phase extraction (SPE) methods while being easier to operate and using less solvent (Wudy et al., 2000; Etter et al., 2006).

In this case, different ratios of *n*-hexane/ethyl acetate (2:3, 1:1, and 3:2, v:v) were chosen to extract steroid hormones from plasma samples. According to the method which was described in previous studies for steroid hormones (Zhang et al., 2017), the extraction efficiency was evaluated by the absolute recovery of steroid hormones from plasma, and a stripped plasma was used for the recovery study (QC3). In this study, we prepared two groups of samples. The plasma was spiked with IS after extraction in the first group. And for the second group, IS was spiked in plasma before extraction. The absolute recovery of plasma steroid hormones was calculated from the comparison of the intensity ratios of two groups. The results showed that the mean extraction efficiency was determined to be ≥83.6% (*n* = 3) by using 1 ml *n*-hexane/ethyl acetate (1:1, v/v).

### 3.4 Method validation

The assay was validated for linearity, analytical sensitivity, accuracy, precision, specificity, and carryover.

#### 3.4.1 Sensitivity and linearity

For each steroid hormone, a 7-point calibration curve was created. The typical linear responses of these steroid hormones, along with the LOD and LLOQ are given in Table 2. Good linearity coefficients (*r* ^2^ > 0.998) were also obtained for most hormones in the required concentration range except for 21-deoxycortisol (*r* ^2^ = 0.9967) and androstenediol (*r* ^2^ = 0.9952).

**TABLE 2:** Retention time, linearity, and sensitivity for each steroid.

Steroid	Retention time (min)	Linearity			Sensitivity (ng/ml)	
		Range (ng/ml)	Equation	*r* ^2^	LoD (S/N ≥ 3)	LLoQ (S/N ≥ 10)
Aldosterone	1.69	0.1–20	*y* = 0.9352*x* − 0.0133	0.9989	0.01	0.05
18OH coryicosterone	1.83	0.1–20	*y* = 0.0719*x* + 0.0031	0.9982	0.05	0.1
Cortisone	2.06	1.5–300	*y* = 0.0479*x* + 0.0082	0.9996	0.1	1.0
Cortisol	2.21	1.5–300	*y* = 0.0448*x* − 0.0078	0.9991	0.2	1.0
21-Deoxycortisol	2.79	0.1–20	*y* = 0.8178*x* + 0.0322	0.9967	0.05	0.1
Corticosterone	3.04	0.1–20	*y* = 0.6865*x* + 0.0066	0.9997	0.01	0.05
11-Deoxycortisol	3.19	0.1–20	*y* = 0.8070*x* + 0.0156	0.9996	0.01	0.05
Androstenedione	3.68	0.1–20	*y* = 0.2282*x* + 0.0027	0.9988	0.01	0.05
Deoxcorticosterone	3.98	0.1–20	*y* = 1.0350*x* + 0.0001	0.9996	0.01	0.05
Testosterone	4.05	0.1–20	*y* = 0.2941*x* − 0.0018	0.9998	0.01	0.05
Androstenediol	4.06	1.5–300	*y* = 0.0045*x* + 0.0087	0.9952	1.0	1.5
DHEA	4.38	15–3,000	*y* = 0.0004*x* + 0.0014	0.9995	4.0	15
17-OHP	4.40	0.1–20	*y* = 0.4351*x* + 0.0037	0.9995	0.01	0.05
17-hydroxy pregnenolone	4.45	1.5–300	*y* = 0.0068*x* + 0. 0034	0.9985	0.5	1.0
Dihydrotestosterone	5.36	0.1–20	*y* = 0.6425*x* − 0.0044	0.9995	0.01	0.05
Progesterone	5.51	0.25–50	*y* = 0.0307*x* + 0.0012	0.9995	0.02	0.1
Pregnenolone	5.57	0.25–50	*y* = 0.0374*x* + 0.0125	0.9991	0.2	1.0

#### 3.4.2 Accuracy and imprecision

We determined the recovery by analyzing charcoal stripped plasma to which we added low, medium, and high standards and is given in Table 3. The samples were analyzed three times, and the recoveries for the steroid hormones ranged from 91.7 to 109.8%.

**TABLE 3:** Recovery for each steroid hormone.

Steroid hormones	Level 1			Level 2			Level 3		
	Spiked amount (ng/ml)	Mean detected (ng/ml)	Mean recovery (%)	Spiked amount (ng/ml)	Mean detected (ng/ml)	Mean recovery (%)	Spiked amount (ng/ml)	Mean detected (ng/ml)	Mean recovery (%)
Aldosterone	0.50	0.48	96.2	2.00	2.09	104.7	8.00	8.36	104.7
18OH corticosterone	0.50	0.50	108.9	5.00	4.70	94.4	20.00	20.20	101.1
Cortisone	1.50	1.40	92.3	30.00	29.60	98.7	150.00	151.90	101.3
Cortisol	7.50	7.48	99.8	30.00	32.13	107.1	120.00	131.81	109.8
21-Deoxycortisol	0.50	0.47	94.0	2.00	2.02	101.2	8.00	8.20	102.5
Corticosterone	0.50	0.47	94.3	2.00	1.95	97.5	8.00	7.63	95.4
11-Deoxycortisol	0.50	0.48	96.5	2.00	2.10	104.8	8.00	8.39	104.8
Androstenedione	0.50	0.54	108.9	2.00	2.18	109.2	8.00	7.64	95.6
11-Deoxycorticosterone	0.50	0.49	97.0	2.00	2.18	109.0	8.00	7.86	98.3
Testosterone	0.50	0.47	93.2	2.00	1.95	97.5	8.00	7.74	96.8
Androstenediol	5.00	5.40	108.9	30.00	28.90	96.3	100.00	106.20	106.2
DHEA	200.00	216.90	108.5	800.00	851.40	106.4	3,200.00	3,168.73	99.0
17α-OHP	0.50	0.46	91.7	2.00	2.01	100.4	8.00	7.89	98.6
17OH-pregnenolone	1.50	1.60	106.7	15.00	14.40	96.2	150.00	142.20	94.9
Dihydrotestosterone	0.50	0.48	96.2	2.00	1.99	99.8	8.00	7.85	98.1
Progesterone	2.50	2.35	94.1	10.00	10.16	101.6	40.00	40.51	101.3
Pregnenolone	7.50	7.49	99.8	30.00	27.82	92.8	120.00	115.73	96.5

Total imprecision was assessed by analyzing three concentration levels QCs in triplicate over 5 days according to CLSI document EP10-A3. As shown in Table 4, the total precision was within the acceptable range (CV < 10%).

**TABLE 4:** Total imprecision for each steroid hormone.

Steroid hormones	QC1		QC2		QC3	
	Concentration (ng/ml)	CV(%), n	Concentration (ng/ml)	CV(%), n	Concentration (ng/ml)	CV(%), n
Aldosterone	0.24	6.92	0.32	5.04	0.49	4.48
18OH corticosterone	0.91	2.22	4.84	2.62	10.06	2.88
Cortisone	1.497	7.55	20.68	1.37	151.94	1.2
Cortisol	8.00	3.50	95.65	2.39	126.57	3.25
21-Deoxycortisol	0.80	3.15	4.92	2.53	10.16	2.09
Corticosterone	0.49	5.81	3.11	2.27	6.43	3.01
11-Deoxycortisol	0.52	1.85	1.02	1.67	4.87	0.92
Androstenedione	0.54	3.77	1.25	6.62	5.87	6.86
11-Deoxcorticosterone	0.17	9.49	0.74	7.25	6.27	8.01
Testosterone	0.47	4.39	0.94	4.12	4.93	4.68
Androstenediol	5.45	4.45	44.08	5.92	108.5	1.39
DHEA	0.45	4.12	228.93	5.65	1,237.99	4.33
17OHP	0.49	3.12	1.77	2.54	5.71	1.89
17OH-pregnenolone	2.16	2.25	12.84	5.67	142.24	1.24
Dihydrotestosterone	0.13	8.14	0.48	5.50	4.51	3.46
Progesterone	2.39	1.64	15.14	1.01	31.79	1.18
Pregnenolone	0.49	9.30	7.88	6.13	79.98	3.71

*n* = 3*3*5 = 45.

#### 3.4.3 Specificity

**FIGURE 3:**
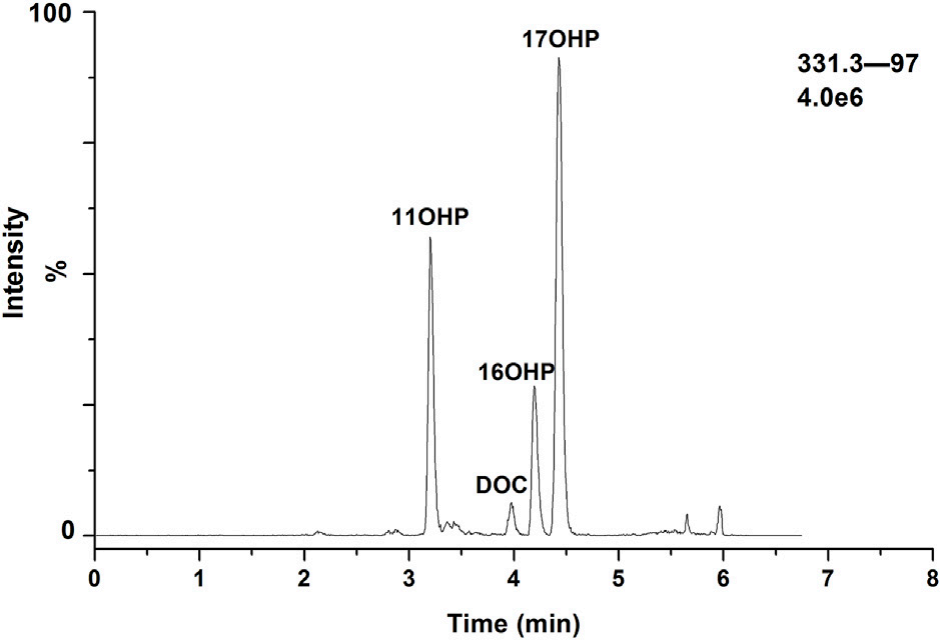
Chromatogram for separation of 17OHP, 11OHP, DOC, and 16OHP.

As the presence of isomers or structural analogues can bias measurements by LC–MS-based methods. As mentioned above, among these structural analogs, 11OHP, 16OHP, and DOC have the same molecular mass as 17OHP. The same situation was also observed in 21-deoxycortisol, corticosterone, and 11-deoxycortisol, which had a molecular weight of 346.46. The molecular weight of aldosterone and cortisone were 360.44. The molecular weight of 18OH-corticosterone and cortisol were 362.46 (Table 5). After optimization, the LC conditions of this method allow complete baseline resolution of these structural analogs, which shares the same multiple reaction monitoring transition, in 7 min (Figures 2, 3).

**TABLE 5:** Steroid hormones used for specificity analysis, including the molecular mass and the concentration used during testing.

Name	Molecular weight	CAS no.	Retention time	Q1 mass (Da)	Q3 mass (Da)
17OHP	330.46	604-09-1	4.40	331.3	97.0
11OHP	330.46	80-75-1	3.23	331.3	97.0
DOC	330.46	64-85-7	3.98	331.3	97.0
16OHP	330.46	438-07-3	4.24	331.3	97.0
21-Deoxycortisol	346.46	641-77-0	2.79	347.2	121.1
Corticosterone	346.46	50-22-6	3.04	347.3	329.0
11-Deoxycortisol	346.46	152-58-9	3.19	347.2	97.0
Aldosterone	360.44	52-39-1	1.69	361.0	343.0
Cortisone	360.44	53-06-5	2.06	361.3	163.1
18OH-corticosterone	362.46	561-65-9	1.83	363.0	121.1
Cortisol	362.46	50-23-7	2.21	363.0	121.0

#### 3.4.4 Carryover

The ratio of needle washing solution was optimized by using the following mixture solution comprised of 50% water, 25% acetonitrile and 25% isopropanol. After injection of the highest concentration of the analyte, no significant carryover was observed.

### 3.5 Clinical application

#### 3.5.1 Multivariate statistical analysis

**FIGURE 4:**
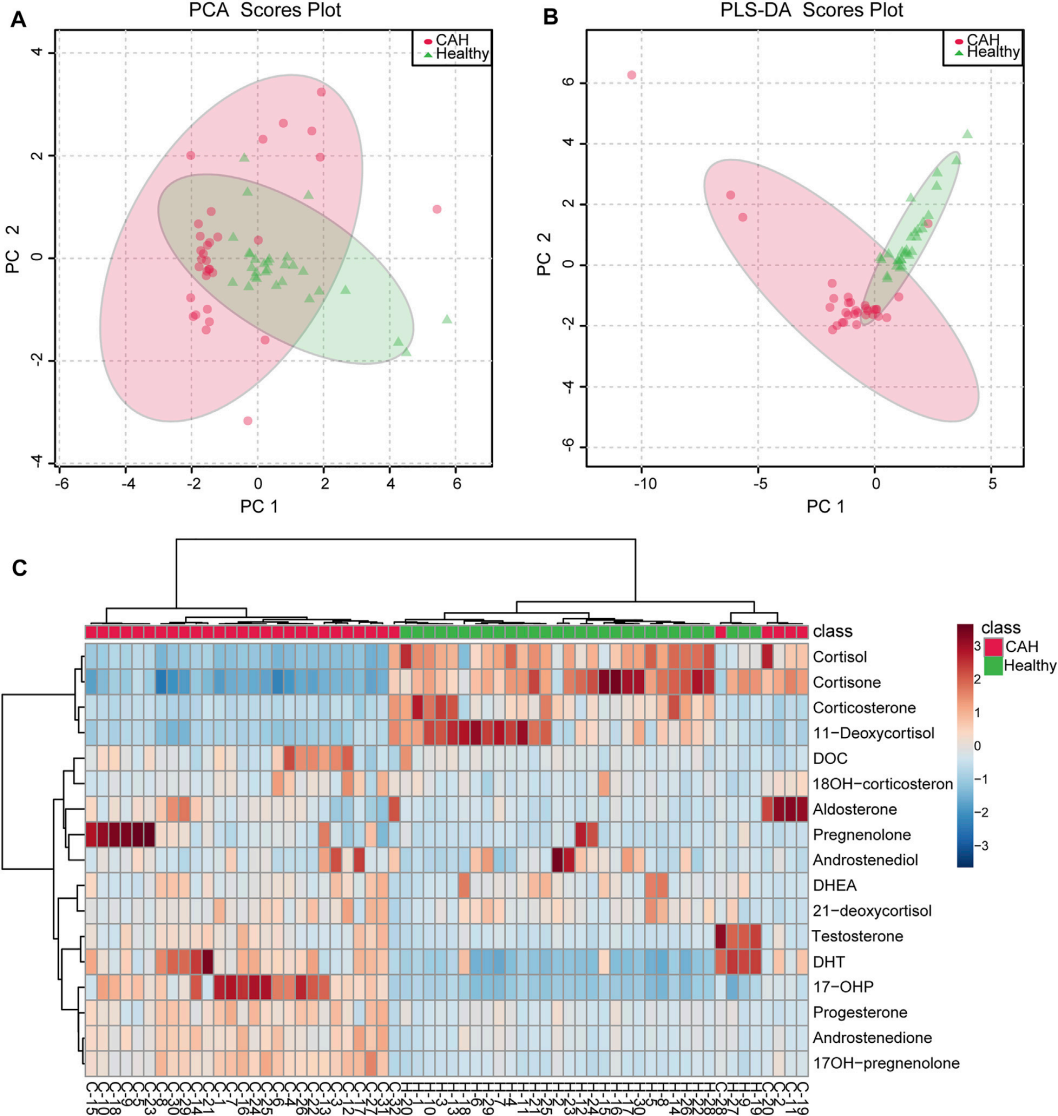
Multivariate statistical analysis for CAH patients (red circle) and healthy volunteers (green triangle). **(A)** PCA score plots of the multivariate data of 17 differential hormones. **(B)** PLS-DA score plot based on 17 differential hormones. **(C)** Heatmap represents for 17 hormones concentration change in CAH patients and healthy volunteers. Red color indicates relative enrichment, whereas blue indicates relative depletion.

Multivariate statistical analysis was performed to determine whether there were significant differences between CAH patients and healthy volunteers. Principal component analysis (PCA) was initially performed, and there was a tendency for group separation between control and CAH. Each group presented clustering status, but some samples were crossed (Figure 4A). Further we established the partial least squares discrimination analysis (PLS-DA) mode on the target metabolomics data for 17 hormones to eliminate interference from various non-experimental factors. As showed in Figure 4B, there was a certain difference between patients and volunteers. The model parameters of R2X and Q2 (cum) are 73.3 and 79.4%, respectively, representing the superior fit and prediction ability of PLS-DA model. In order to more intuitively see the differences in plasma levels of 17 hormones in 32 CAH patients and 30 healthy volunteers, a heat map analysis was conducted, as shown in Figure 4C. It can be seen from the figure that CAH patients can be clearly distinguished from healthy controls, and the levels of 17 hormones are significantly different between the two groups. Among them, CAH2, CAH11, CAH19, CAH20, and CAH28 were intersected with the healthy group. We speculated that the above samples were from CAH patients after treatment, and that the patients had recovered when we took the samples.

#### 3.5.2 Potential biomarkers identification

**FIGURE 5:**
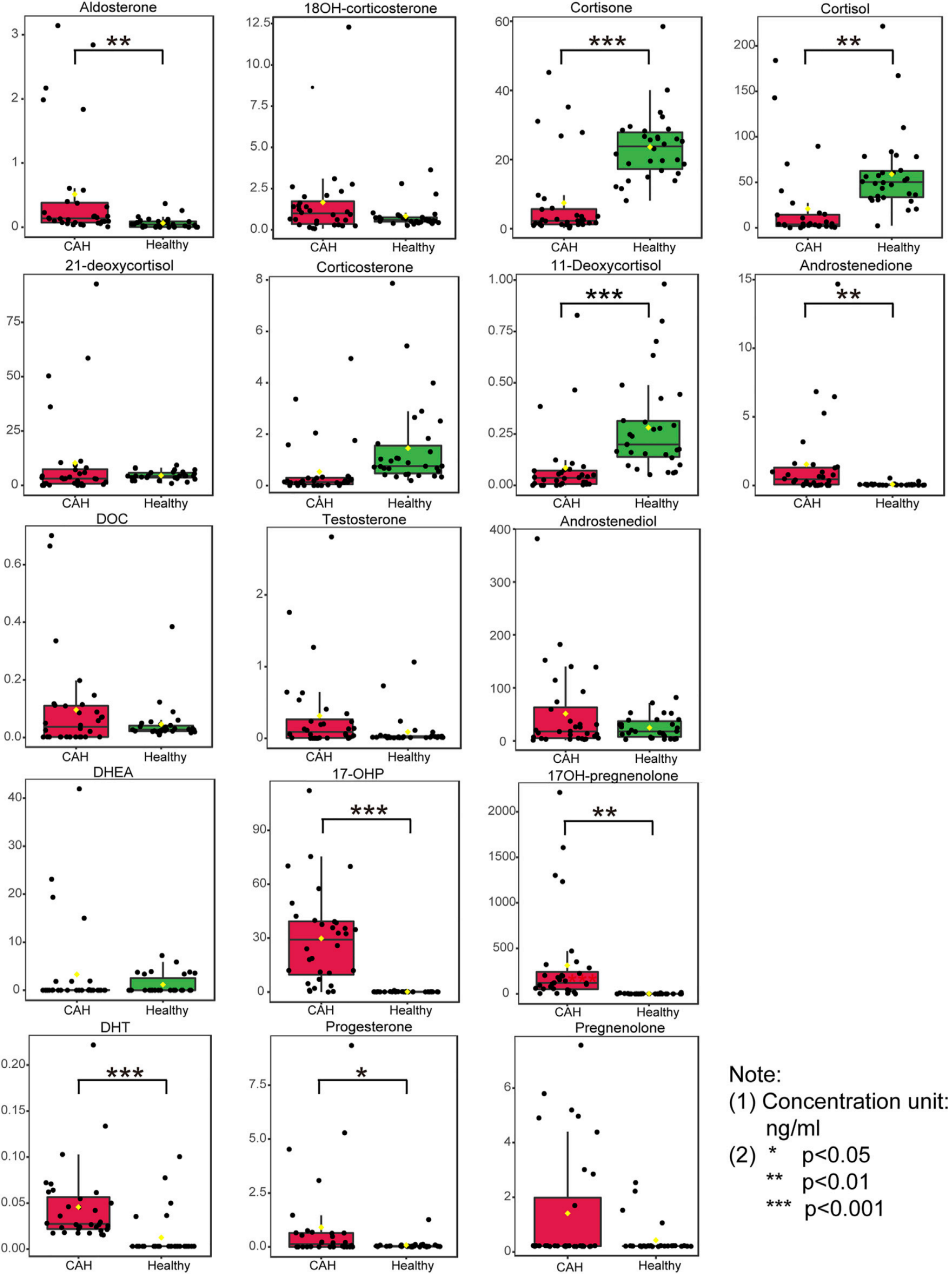
Boxplots of the 17 hormones concentration in plasma specimens from 32 congenital adrenal hyperplasia (CAH) patients and 30 healthy volunteers. (*p < 0.05, **p < 0.01, ***p < 0.001)

**FIGURE 6:**
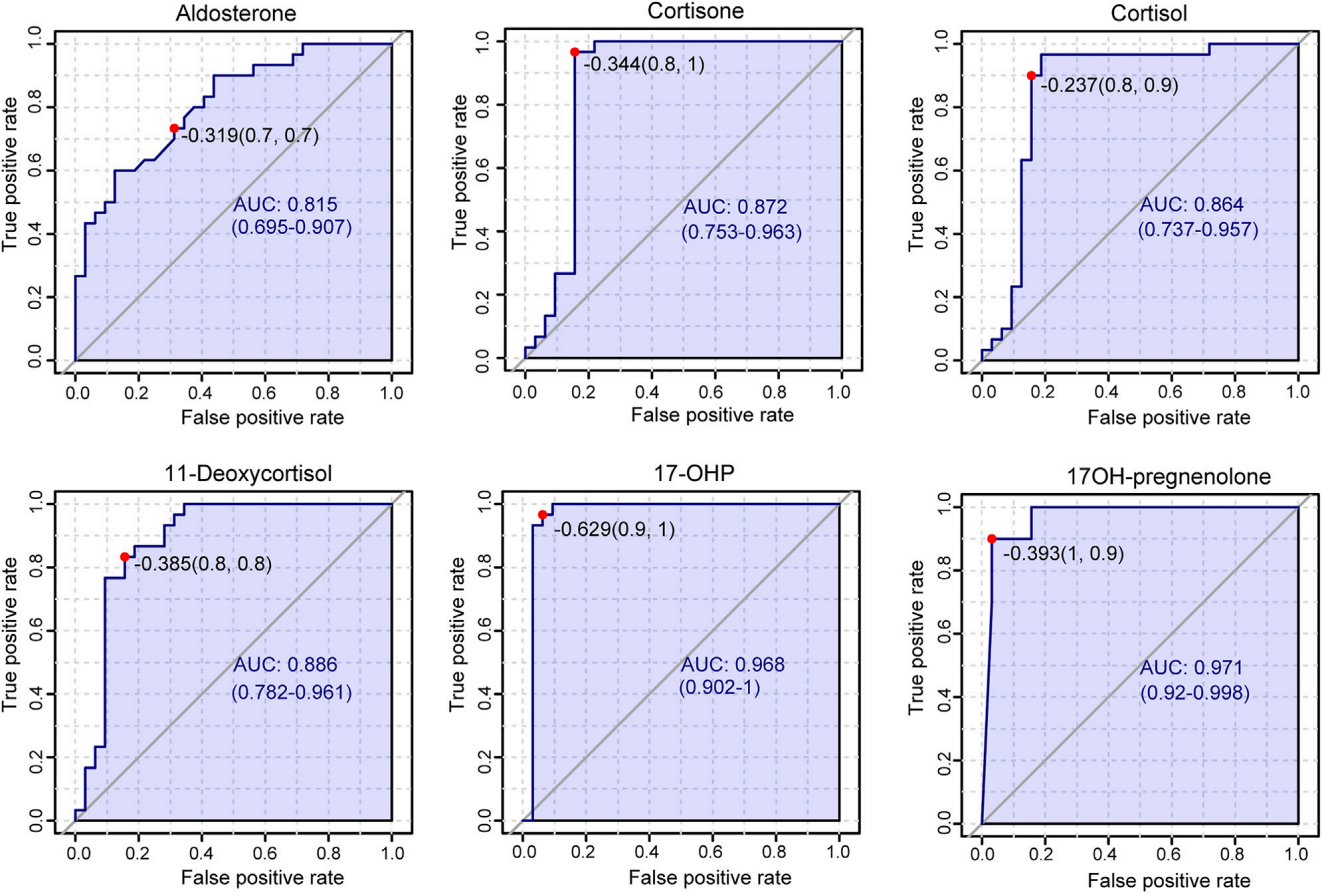
Results of receiver operating characteristic curve (ROC) analysis.

Plasma contents of 17 hormones in the CAH group and the healthy control group were statistically analyzed, and the mean ± standard (SD) deviation was used to represent the contents in each group. At the same time, SPSS software was used for independent sample *t* test to analyze the statistical difference of each index between the two groups. The fold change between the two groups was analyzed by multiple transformations, and the trend of increase/decrease was described. Explicit results are given in Table 6. In addition, plasma levels of 17 hormones in the CAH group and the control group were also presented in the form of box plots, as shown in Figure 5. Combining multivariate statistics and university statistics, we screened five differential hormones based on VIP >1.0 with *p* < 0.05 and large fold change between the two groups, including 17OHP, cortisol, 11-deoxycortisol, DHEA, cortisone. The fold change of 17OH-pregnenolone between the two groups is 198.372, and it is a substantial change, so we focused on this hormone. Discriminate models based on these six differential hormones show great potential for identifying CAH patients from healthy people, so these six differential metabolites could serve as potential biomarkers for CAH. To further understand the predictive power of the six potential biomarkers mentioned above, ROC curve analysis was performed. Subsequently, ROC curves of these six candidates were exploited based on the results of the area under the curve (AUC), the sensitivity, and specificity at best cutoff points, as shown in Figure 6. Well, depending on the chart, it is obvious that 17OHP and 17OH-pregnenolone achieved better AUC values, sensitivity, and specificity, and more powerful diagnostic potential.

**TABLE 6:** Statistical table of plasma levels of 17 hormones in CAH group and healthy control group (ng/ml).

No.	Name	Group	Number	Mean ± SD/ (ng/ml)	*p* Value	VIP score	Fold change	Variation trend
1	Aldosterone	CAH	32	0.514 ± 0.860	*p* < 0.01	1.619	8.044	↑
		Healthy	30	0.064 ± 0.083^a^				
2	18OH-corticosterone	CAH	32	1.666 ± 2.495	*p* > 0.05	0.802	2.006	↑
		Healthy	30	0.830 ± 0.740				
3	Cortisone	CAH	32	7.445 ± 11.780	*p* < 0.001	1.348	0.315	↓
		Healthy	30	23.632 ± 9.817				
4	Cortisol	CAH	32	20.968 ± 42.717	*p* < 0.01	1.145	0.355	↓
		Healthy	30	58.999 ± 43.514				
5	21-DF	CAH	32	10.306 ± 20.514	*p* > 0.05	0.935	2.276	↑
		Healthy	30	4.527 ± 2.018				
6	Corticosterone	CAH	32	0.537 ± 1.094	*p* > 0.05	0.954	0.219	↓
		Healthy	30	2.452 ± 6.175				
7	11-deoxycortisol	CAH	32	0.085 ± 0.170	*p* < 0.001	1.283	0.301	↓
		Healthy	30	0.282 ± 0.233				
8	Androstenedione	CAH	32	1.542 ± 2.998	*p* < 0.01	0.867	20.423	↑
		Healthy	30	0.076 ± 0.101				
9	DOC	CAH	32	0.095 ± 0.171^a^	*p* > 0.05	0.993	2.053	↑
		Healthy	30	0.046 ± 0.068^a^				
10	Testosterone	CAH	32	0.315 ± 0.598	*p* > 0.05	0.75	3.479	↑
		Healthy	30	0.091 ± 0.228				
11	Androstenediol	CAH	32	51.384 ± 79.081	*p* > 0.05	0.667	2.078	↑
		Healthy	30	24.731 ± 21.179				
12	DEHA	CAH	32	3.289 ± 9.084	*p* > 0.05	0.66	2.831	↑
		Healthy	30	1.162 ± 2.080				
13	17-OHP	CAH	32	29.830 ± 26.377	*p* < 0.001	1.229	205.157	↑
		Healthy	30	0.145 ± 0.156				
14	17OH-pregnenolone	CAH	32	312.734 ± 521.21	*p* < 0.01	0.877	198.372	↑
		Healthy	30	1.577 ± 3.718				
15	Dihydrotestosterone	CAH	32	0.046 ± 0.042^a^	*p* < 0.001	0.872	4.532	↑
		Healthy	30	<LLOQ				
16	Progesterone	CAH	32	0.907 ± 2.000	*p* < 0.05	0.865	11.306	↑
		Healthy	30	0.080 ± 0.226				
17	Pregnenolone	CAH	32	2.059 ± 5.292	*p* > 0.05	0.454	8.385	↑
		Healthy	30	0.246 ± 0.673				

a The median is below the linear range, but near or greater than LLoQ.

These results further revealed that measuring multiple adrenal corticosteroid hormones simultaneously could provide a more comprehensive monitoring of hormone metabolism, which can not only be used for CAH caused by 21-hydroxylase, but also has potential diagnostic and auxiliary diagnostic value for CAH caused by other causes. The results confirmed that the combined measurement of multiple hormones could indeed improve the diagnostic efficiency of CAH.

## 4 Conclusion

A sensitive and accurate isotope-dilution UHPLC-MS/MS method was developed and it demonstrated that it can simultaneously quantify 17 endogenous adrenal corticosteroid hormones in human plasma without derivatization. Liquid-liquid extraction was used to extract and concentrate steroid hormones from plasma samples to achieve the required sensitivity. The isomers and structure analogues, such as 17OHP (11OHP, 16OHP, and DOC), 21-deoxycortisol (corticosterone and 11-deoxycortisol), aldosterone (cortisone), and 18OH-corticosterone (cortisol) were successfully separated by optimization of the ESI source, MRM, and LC conditions. We have also fully validated the method, which achieved good results in terms of sensitivity, specificity, linearity, accuracy, and precision. The optimized method was successfully applied to measure 17 endogenous adrenal corticosteroid hormones in plasma specimens from 32 congenital adrenal hyperplasia (CAH) patients and 30 healthy volunteers. The results showed that measuring steroid hormones simultaneously and establishing the combined diagnosis model of multiple hormones could improve the diagnostic efficiency of CAH. We anticipate that the approach for quantitation of endogenous adrenal corticosteroid hormones described here will ultimately be applied to clinical biological samples. It should be noted that this method does not distinguish between endogenous and exogenous hormones, so drugs or other exogenous hormones may cause inaccurate results.

## Data Availability

The original contributions presented in the study are included in the article/Supplementary Material; further inquiries can be directed to the corresponding authors.
