# Supplementary material for: Simultaneous quantitation of 17 endogenous adrenal corticosteroid hormones in human plasma by UHPLC-MS/MS and their application in congenital adrenal hyperplasia screening
Source: Front Chem. 2022 Aug 11;10:961660. doi: 10.3389/fchem.2022.961660 (PMC9403142; doi:10.3389/fchem.2022.961660)
Supplement: Supplementary file 1 [file DataSheet1.docx]

**Supporting information for**

**Simultaneous quantitation of** **17 endogenous adrenal corticosteroid hormones in human** **plasma by** **UHPLC-MS/MS and application in congenital adrenal hyperplasia screening**

**Table of Contents:**

**Table S1. The** **basic information on these subjects**

|  | **Control (Health)** | **Congenital adrenal hyperplasia (CAH)** |
| --- | --- | --- |
|  | (N=30) | (N=32) |
| Sex |  |  |
| Male-no.(%) | 11（37%） | 16(50%) |
| Female-no.(%) | 19（63%） | 16(50%) |
| Age-year |  |  |
| Mean±SD | 4.7 ± 4.0 | 7.0 ± 3.5 |
| Range | 0.3-11 | 0.3-13 |

**Figure of Contents:**

Figure S1. Spectra of parental ions and their product ions as a result of collisional activation of the 17 endogenous adrenal corticosteroid hormones.

1. Aldosterone

2. 18OH-corticosterone

3. Cortisone

4. Cortisol

5. 21-Deoxycortisol

6. Corticosterone

7. 11-deoxycortisol

8. Androstenedione

9. 11-deoxycorticosterone

1. Testosterone

11. Androstenediol(255.1-159.1)

12. DHEA

1. 17-OHP

14. 17OH-pregnenolone

15. DHT

1. Progesterone

17. Pregnenolone
